# Recombinant human G6PD for quality control and quality assurance of novel point-of-care diagnostics for G6PD deficiency

**DOI:** 10.1371/journal.pone.0177885

**Published:** 2017-05-26

**Authors:** Maria Kahn, Nicole LaRue, Changcheng Zhu, Sampa Pal, Jack S. Mo, Lynn K. Barrett, Steve N. Hewitt, Mitchell Dumais, Sandra Hemmington, Adrian Walker, Jeff Joynson, Brandon T. Leader, Wesley C. Van Voorhis, Gonzalo J. Domingo

**Affiliations:** 1 PATH Diagnostics Group, Seattle, Washington, United States of America; 2 Center for Emerging and Re-emerging Infectious Diseases (CERID), Division of Allergy and Infectious Diseases, Department of Medicine, University of Washington, Seattle, Washington, United States of America; 3 Mologic Ltd, Bedford Technology Park, Thurleigh, Bedfordshire, United Kingdom; Centro de Pesquisas Rene Rachou, BRAZIL

## Abstract

**Background:**

A large gap for the support of point-of-care testing is the availability of reagents to support quality control (QC) of diagnostic assays along the supply chain from the manufacturer to the end user. While reagents and systems exist to support QC of laboratory screening tests for glucose-6-phosphate dehydrogenase (G6PD) deficiency, they are not configured appropriately to support point-of-care testing. The feasibility of using lyophilized recombinant human G6PD as a QC reagent in novel point-of-care tests for G6PD deficiency is demonstrated.

**Methods:**

Human recombinant G6PD (r-G6PD) was expressed in *Escherichia coli* and purified. Aliquots were stored at -80°C. Prior to lyophilization, aliquots were thawed, and three concentrations of r-G6PD (representing normal, intermediate, and deficient clinical G6PD levels) were prepared and mixed with a protective formulation, which protects the enzyme activity against degradation from denaturation during the lyophilization process. Following lyophilization, individual single-use tubes of lyophilized r-G6PD were placed in individual packs with desiccants and stored at five temperatures for one year. An enzyme assay for G6PD activity was used to ascertain the stability of r-G6PD activity while stored at different temperatures.

**Results:**

Lyophilized r-G6PD is stable and can be used as a control indicator. Results presented here show that G6PD activity is stable for at least 365 days when stored at -80°C, 4°C, 30°C, and 45°C. When stored at 55°C, enzyme activity was found to be stable only through day 28.

**Conclusions:**

Lyophilized r-G6PD enzyme is stable and can be used as a control for point-of-care tests for G6PD deficiency.

## Background

In 2015, malaria caused an estimated 214 million cases and 438,000 deaths [1]. While *Plasmodium (P*.*) falciparum* is the major source of the malaria burden, *P*. *vivax* is more widespread and, in many regions, especially those approaching elimination, is the main cause of malaria. In contrast to *P*. *falciparum*, *P*. *vivax* can lay dormant in the human liver and relapse weeks to months after clearance of the parasites circulating in the blood with typical schizonticidal drugs [2–5]. The relapses are an iterative source of deteriorating health in the infected patient and of ongoing transmission of the parasite. The only drugs available that cure patients of the parasite are 8-aminoquinoline–based drugs such as primaquine. The dosage required for these drugs to be effective curative agents for *P*. *vivax* infection may cause severe hemolysis in patients with glucose-6-phosphate dehydrogenase (G6PD) deficiency [6, 7]. G6PD deficiency is one of the more common genetic traits—often with higher prevalence in malaria at-risk populations [8–11]. Providing safe access to curative drugs for *P*. *vivax* in many populations requires testing for G6PD deficiency at the point-of-care where people seek malaria treatment [4, 5]. The G6PD fluorescent spot test (FST) for G6PD deficiency is perhaps the most typical standard of care, but it is too complex to implement in remote settings with limited clinical laboratory access. In recent years there has been an effort to advance the development of point-of-care tests for G6PD deficiency that can be made available closer to the malaria patient.

A large gap for the implementation of point-of-care G6PD testing is the availability of reagents to support quality control (QC) of G6PD products along the supply chain from the manufacturer to the end user. The absence of similar reagents has also been a challenge in the implementation of human immunodeficiency virus (HIV) and malaria rapid diagnostic tests (RDTs) [12–15]. Key aspects to the utility of these reagents are stability and unitized packaging [16]. Although reagents and systems exist to support QC of laboratory screening tests for G6PD, they are not formulated or packaged adequately to support programmatic quality assurance (QA) programs for point-of-care G6PD tests. These reagents could support a framework for a sustainable QC/QA system at country level to support robust point-of-care G6PD testing for *P*. *vivax* radical cure.

## Methods

### Expression and purification of recombinant G6PD

Recombinant human G6PD (r-G6PD) was cloned and expressed in *Escherichia* (*E*.*) coli* cultures as described by Wang et al [20]. Cell pellets were harvested and stored at -80°C. Cell pellets were thawed and added to 150 to 200 mL of equilibration buffer (0.1 M Tris/HCl, 5 mM EDTA, 5 mM β-mercaptoethanol, pH 7.3) and 1 mM TCEP, 250 μg/mL AEBSF. Cells were lysed by sonicating for 5 minutes (30-second bursts with 1-minute rest intervals). This solution was centrifuged at 29,800 RCF for 1 hour. The supernatant (containing soluble G6PD) was run over a 2’, 5’ ADP Sepharose column and then washed with 150 to 300 mL of equilibration buffer. Elution of G6PD was done with 40 μM NADP^+^ in equilibration buffer. Concentrating the protein was done by centrifuging at 4,300 RCF with a size-exclusion filter. Protein purity was checked with SDS-PAGE and concentration was measured with UV-Vis spectrophotometry. Purified protein was stored at -80°C.

### Lyophilization of recombinant G6PD

Recombinant human G6PD (r-G6PD) was expressed in *E*. *coli* and purified. The purified r-G6PD was stored in aliquots in protein storage buffer (PSB) at -80°C. The protein storage buffer contained 100 mM Tris-HCl, 5 mM EDTA, 5 mM β-mercaptoethanol, 40 μM NADP+, pH 7.3. All reagents were purchased from Sigma-Aldrich (St. Louis, MO, USA). Three concentrations of r-G6PD were selected for the lyophilization study. Each concentration was combined with a 2x sucrose/mannitol formulation (final concentration: mannitol 3% and sucrose 1%) and subjected to lyophilization. For lyophilization, 50 μL of formulated r-G6PD was aliquoted into 0.2 mL PCR strip tubes and placed in the lyophilizer with the lids open. Lyophilization was performed in a Millrock Laboratory Freeze Dryer (Millrock Technology, Kinston, NY, USA). The freeze cycle started with the samples being held at 4°C for 3 hours, then to -45°C for 6 hours, to -10°C for 2 hours, with completion at -45°C for 5 hours. The vacuum was set at 60 mTorr. During the primary dry, the samples were held at -25°C for 25 hours, followed by a hold at 4°C for 8 hours. During the secondary dry, the samples were placed at 4°C for 60 minutes. After freeze drying, the tubes were back-filled with nitrogen gas and capped. Samples were then individually packaged, with a desiccant pack, and sealed under humidity- and temperature-controlled conditions.

### Assays for G6PD activity

To measure G6PD activity in the lyophilized samples, the tubes were removed from the individually sealed packages and spun down briefly in a mini centrifuge to ensure that all dried down sample was in the bottom of each tube. Deionized water (100 μL) was added to each tube, mixed by flicking, and left on ice for 10 minutes to ensure that the sample was completely rehydrated. The tubes were then vortexed briefly and spun before use. Samples were left on ice and were used within one hour.

The rehydrated r-G6PD was assayed using the Trinity Biotech quantitative G6PD reference assay (catalog number 345-B; Trinity Biotech PLC, Bray, Ireland). The r-G6PD was characterized for G6PD activity in duplicate according to the manufacturer’s instructions`Normal, intermediate, and deficient Trinity Biotech assay controls (catalog numbers G6888, G5029, and G5888, respectively) were run using the same method on each day of testing.

Briefly, 10 μL of rehydrated r-G6PD was added to 1 mL of reagent and incubated at room temperature for 5 minutes. Two milliliters (2 mL) of substrate was added to the solution and mixed by inversion. Then 1 mL of the mixture was aliquoted into each of two ultraviolet (UV)-transparent disposable cuvettes (catalog number 47727–024; Brand Co., Wertheim, Germany). The duplicate cuvettes were incubated at 30°C in a water bath for 5 minutes. Enzyme activity was determined using a temperature-regulated spectrophotometer (UV-1800 Shimadzu; Shimadzu Scientific Instruments, Columbia, MD, USA) set at 30°C by measuring the change in rate in absorbance at 340 nm over 5 minutes. G6PD activity values were calculated in units per deciliter using the values in the Trinity assay (catalog number 345-B; Trinity Biotech PLC, Bray, Ireland) insert. The values were measured at 30°C and no temperature correction factor was applied.

### Trinity biotech fluorescent spot test

The widely used qualitative fluorescent spot test (FST) for G6PD deficiency was used to demonstrate the utility of the lyophilized recombinant G6PD for confirming the performance of the test. The recombinant controls and the Trinity normal, intermediate, and deficient controls were tested using Whatman No. 1 filter paper (catalog number 1001–150) with the Trinity qualitative G6PD FST kit (catalog number 203-A; Trinity Biotech) according to the manufacturer’s instructions. The method detects the fluorescence of NADPH, which is proportional to G6PD activity, under long-wave UV light (365 nm). Briefly, 5 μL of rehydrated r-G6PD was added to 200 μL of the reagent mixture and spotted onto filter paper at time zero. Each sample was incubated at 37°C and spotted again after 5 and 10 minutes of incubation. Fluorescence was observed for the three time points after samples had dried.

### Qualitative G6PD rapid diagnostic test

**Fig 1:**
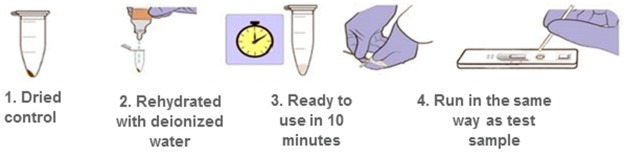
Workflow for the use of the lyophilized control with a glucose-6-phosphate dehydrogenase (G6PD) rapid diagnostic test.

A novel qualitative rapid diagnostic test (RDT) for G6PD deficiency currently under development was used to demonstrate the utility of the lyophilized recombinant G6PD for confirming the performance of a point-of-care test. This test relies on antibodies generated to selected epitopes on the G6PD enzyme. Recombinant entire human G6PD enzyme was used to generate both polyclonal antibodies and monoclonal antibodies. The assay combines the features of a specific target immune capture assay with the detection of G6PD activity by a visual result that is directly proportional to enzyme activity. This test is a two-step process. In the first step, the G6PD is concentrated by the antibody; during the second step the enzyme activity is detected by the development of a precipitating formazan substrate based on NADPH-driven dye reduction as reported previously [21, 22]. Recombinant G6PD was assayed on this qualitative point-of-care RDT. Testing was carried out using the r-G6PD standards, which were prepared to deliver enzyme activity equivalent to normal, intermediate, and deficient G6PD levels, similar to the Trinity controls. The lyophilized controls were rehydrated according to the method above, and 1 μL of reconstituted r-G6PD was added to the sample well. Then 35 μL of buffer was added to the buffer well, after 5minutes, the cassette was closed, and 10 minutes later the result was read. The intensity of the test and the control lines were measured with a small hand-held reader (Fig 1).

### Stability study of lyophilized recombinant G6PD

Since many point-of-care diagnostic assays for G6PD activity will be performed in remote areas, most likely where temperatures and humidity are high, it was necessary to understand the stability of lyophilized r-G6PD. A stability study was initiated to look at different storage temperatures over time. Aliquots of r-G6PD were generated at three concentrations that corresponded to normal, intermediate, and deficient levels of G6PD activity, as determined using the Trinity Biotech quantitative assay. Lyophilized samples were stored at -80°C, 4°C, 30°C, 45°C, and 55°C. At designated times, one aliquot from each concentration was assayed for activity using the Trinity Biotech quantitative assay.

### Reconstituted stability of lyophilized r-G6PD

Since it was found that the shelf life of the reconstituted lyophilized r-G6PD was reliable for one hour post rehydration, different conditions were tested to prolong the shelf life. A study was performed to determine if the diluent used in reconstituting the samples and/or the storage temperature of the rehydrated samples would extend the shelf life of the reconstituted r-G6PD. While the standard protocol is to rehydrate the samples with deionized (DI) water, for this study, in addition to DI water, samples were reconstituted in protein storage buffer (PSB). The samples were tested under four conditions using three concentrations of lyophilized r-G6PD over time. The r-G6PD samples were rehydrated in PSB or DI water and kept at room temperature or on ice after reconstitution. G6PD activity was measured under all four conditions (PSB on ice, PSB at room temperature, DI water on ice, and DI water at room temperature). G6PD activity was measured at time 0 (immediately following rehydration), 1 hour, 2 hours, 4 hours, 6 hours, 8 hours, and 24 hours, using the Trinity Biotech quantitative assay (method described above). At each time point, three concentrations were assessed: normal, high intermediate, and intermediate. For this study, a high-intermediate concentration of r-G6PD was used instead of the deficient to follow change in G6PD activity.

## Results

### Lyophilization of r-G6PD enzyme

Among the desired characteristics for freeze-dried products is an intact cake that is uniform in color, indicating adequate dryness and porosity. Typical combinations of sugars in varying concentrations will contribute to successful lyophilization. In this study, the r-G6PD protein was combined with a formulation of 3% mannitol and 1% sucrose (final concentration) and then subjected to lyophilization. The result was a white, compact, intact pellet that was easily resuspended with DI water or PSB, with no visible aggregates. The formulated recombinant G6PD enzyme were packaged in 0.2 mL PCR strip tubes for resuspension into 100 μL of diluent to support single-use applications.

### Titration of r-G6PD

**Fig 2:**
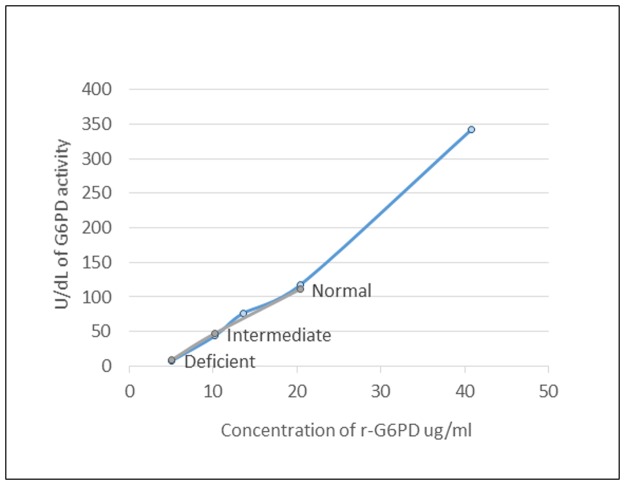
Titration of lyophilized human recombinant glucose-6-phosphate dehydrogenase (r-G6PD). Lyophilized human r-G6PD (blue line) was titrated and tested in the Trinity Biotech quantitative assay alongside the Trinity controls (grey line). From these data, three lyophilized concentrations were chosen to represent normal, intermediate, and deficient enzyme activity.

Initially, the lyophilized r-G6PD was reconstituted with deioinized water and titrations were performed to determine which concentrations had G6PD activities most similar to the activity levels generated with the Trinity Biotech quantitative assay controls (normal, intermediate, and deficient enzyme activities). Fig 2 shows a titration of G6PD activity comparing the three controls from the Trinity assay and the titration of the lyophilized r-G6PD controls. Recombinant G6PD having comparative levels of G6PD activity to the Trinity controls were chosen for further study. The three concentrations of lyophilized r-G6PD that were chosen to be used in this study were 20.4 μg/mL, 10.2 μg/mL and 5.1 μg/mL.

### Stability study

**Fig 3:**
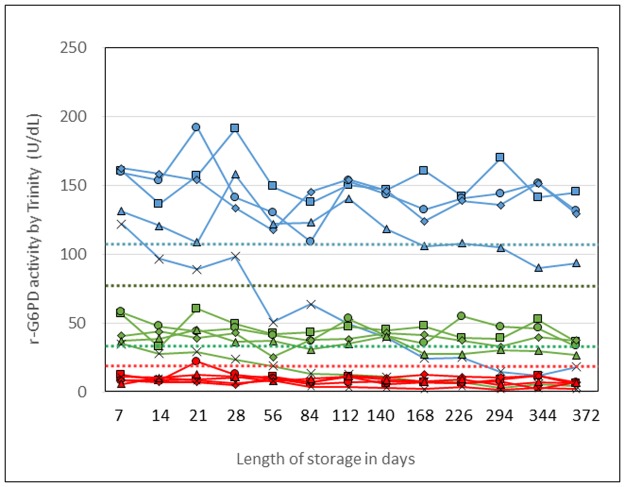
Stability of lyophilized human recombinant glucose-6-phosphate dehydrogenase (r-G6PD) reagent panel: Normal (blue), intermediate (green), and deficient (red). Lyophilized panel of three concentrations of human r-G6PD were stored for 12 months at five different temperatures: -80°C filled squares, 4°C filled circles, 30°C filled diamonds, 45°C filled triangles, and 55°C crosses. Activity was measured using the Trinity Biotech quantitative assay. The graph shows the stability of the panel over time. Stability starts to decline after approximately one month at a high temperature of 55°C. Thresholds are shown to indicate whether each measurement is still within an acceptable range of G6PD enzyme activity based on the ranges of Trinity controls: lower acceptable normal (blue dotted line), upper intermediate (black dotted line), lower intermediate (green dotted line), and upper deficient (red dotted line).

A study was initiated to determine the stability of r-G6PD post-lyophilization by monitoring G6PD activity over time (Fig 3). Three concentrations of recombinant G6PD were lyophilized in individual tubes and stored in sealed packages with desiccant at five temperatures (-80°C, 4°C, 30°C, 45°C, and 55°C).

At times indicated, individual vials of each concentration were removed from each temperature, rehydrated, and assayed for G6PD by the Trinity Biotech quantitative assay. Recombinant G6PD was stable for more than one year when stored at -80°C, 4°C, and 30°C. At 45°C, r-G6PD began to show decline in activity after day 140. At 55°C, the activity of r-G6PD began to decline after day 28 and showed little to no activity after day 168.

### Reconstituted stability of lyophilized r-G6PD

**Fig 4:**
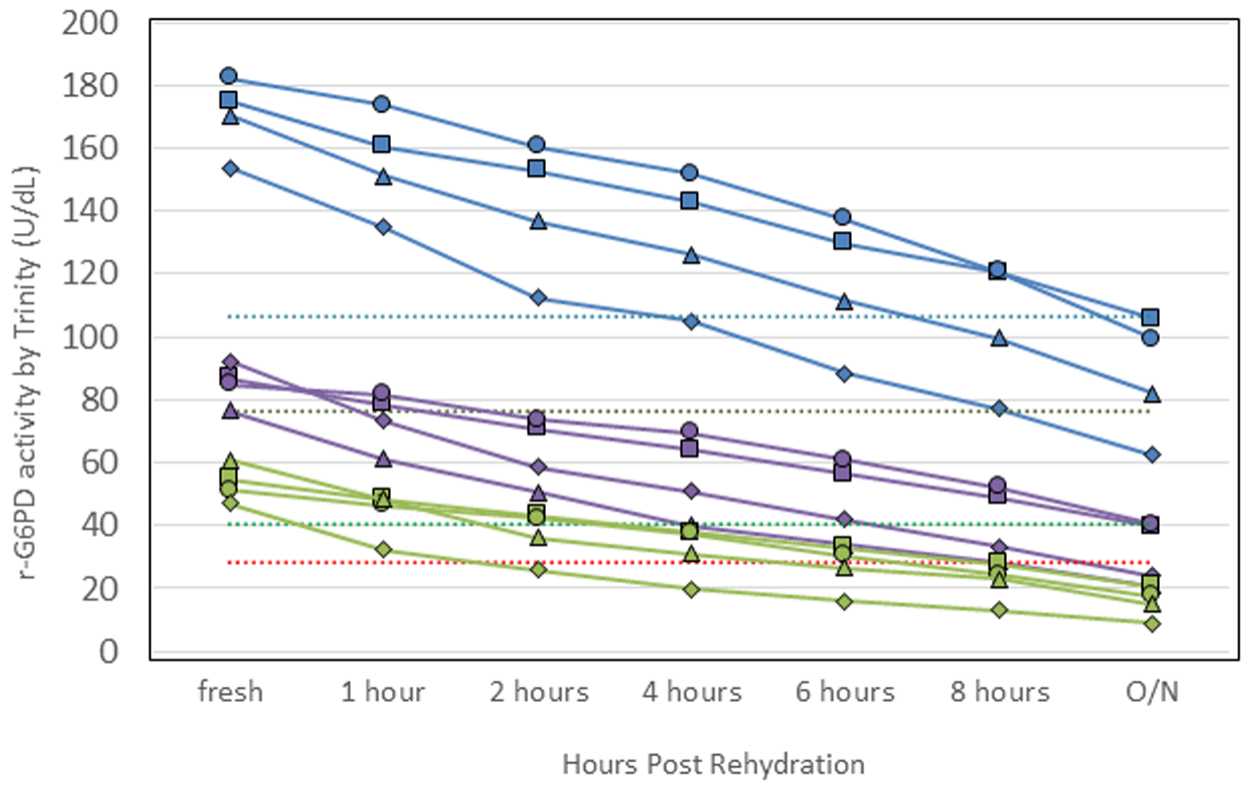
Stability of the reconstituted lyophilized recombinant glucose-6-phosphate dehydrogenase (r-G6PD). Lyophilized controls were reconstituted under four conditions to determine stability after rehydration. Three concentrations were assessed: normal (blue), high intermediate (purple), and intermediate (green). The controls were rehydrated in protein storage buffer (PSB) or deionized (DI) water and kept on ice or at room temperature after rehydration: PSB on ice (filled squares), PSB at room temperature (filled circles), DI water on ice (filled triangles), and DI water at room temperature (filled diamonds). Activity was measured on the Trinity Biotech quantitative assay at time zero, 1 hour, 2 hours, 4 hours, 6 hours, 8 hours, and overnight 24 hours later. Thresholds are shown to indicate whether each measurement is still within an acceptable range of G6PD enzyme activity based on the ranges of Trinity controls: lower acceptable normal (blue dotted line), upper intermediate (black dotted line), lower intermediate (green dotted line), and upper deficient (red dotted line).

A minimal and optimal requirement in the target product profile (TPP) for quality control reagents, called for an extension of shelf life of the reconstituted controls. A study was conducted to assess whether this requirement could be met with the current formulations. The lyophilized controls were reconstituted under four different conditions to determine stability after rehydration. Three concentrations were evaluated: normal, high intermediate, and intermediate. Thresholds based on the accepted activity—of the Trinity controls illustrate whether each r-G6PD activity measurement is still within an acceptable G6PD activity range, see Fig 4. The overall result indicated that, once rehydrated, all controls showed a steady decline in activity over time. Controls reconstituted in PSB showed slightly less decline when compared to controls in water and were able to stay within the expected range for a longer period of time. Storage temperature upon rehydration did not seem to have an effect on stability.

### Performance of r-G6PD controls on novel qualitative tests for G6PD deficiency

**Fig 5:**
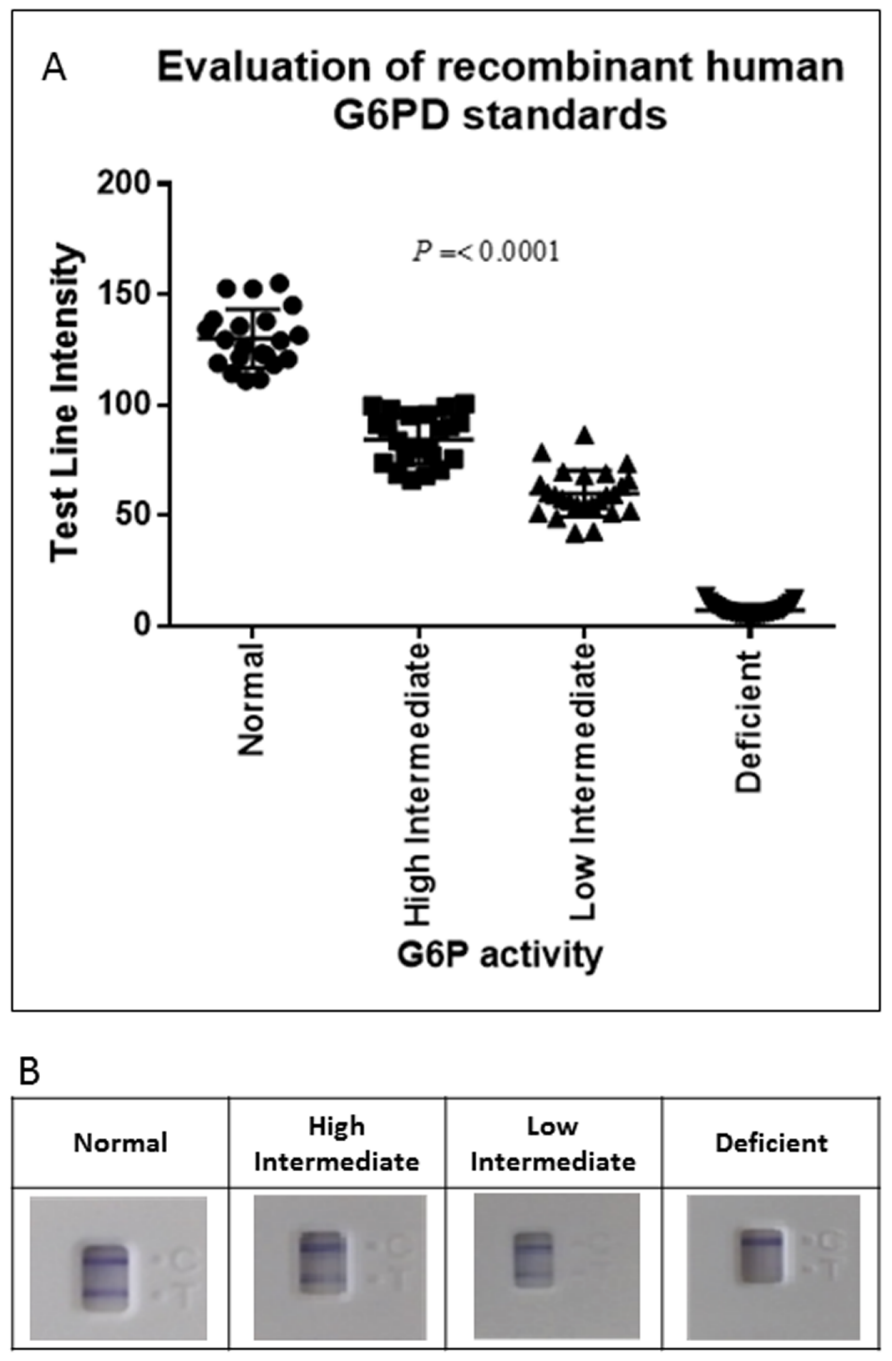
Performance of the positive controls on a qualitative glucose-6-phosphate dehydrogenase (G6PD) test. **A.** Evaluation of human recombinant glucose-6-phosphate dehydrogenase (r-G6PD) standards. The lyophilized r-G6PD controls were tested in the newly developed rapid diagnostic test for G6PD. Line intensity versus G6PD activity was generated using over 20 replicates for each enzyme control. The data show that there are significant differences when comparing intermediate and deficient results to normal. B. Representative images of visual results with r-G6PD standards. Visual results presented here show that the control lines gave similar output across the standards tested. Differences were seen between all groups however, the intermediate high and low test line intensities were similar to the trained eye.

For assessment of the qualitative prototype, currently under development, testing was carried out using freshly lyophilized recombinant human G6PD standards, prepared to deliver assay activity equivalent to normal, intermediate and deficient G6PD levels. The Trinity Biotech quantitative assay was used as the comparative assay and to ensure each lyophilized sample had an adequate level of G6PD activity. The test-line intensities were determined using a low-cost hand-held reader. The results in Fig 5A demonstrate that when the line intensity is plotted versus G6PD activity, the assay demonstrated differences in enzyme activity between normal, intermediate, and deficient groups. The descriptive statistics show differences in the mean values for each standard, but the breakdown does indicate overlap between the high and low intermediate groupings (Table 1). Dunnets multiple comparison test (to compare intermediate and deficient results to normal) did confirm significant differences when compared to the normal G6PD standards. In Fig 5B, the actual results of the tests demonstrates that the control line gave similar outputs across the standards tested. Even with poor image capture and resolution, the visual results demonstrate the clear observable differences between the normal, intermediate and deficient standards.

**Table 1:** Descriptive statistics for data illustrated in Fig 6. Testing of the rapid diagnostic test with recombinant human glucose-6-phosphate dehydrogenase (G6PD) standards, with test-line intensities being measured using a low-cost hand-held reader. Mean difference in readings and lower to upper bounds for a 95% confidence interval (95% CI) as determined by the Dunnetts multiple comparisons test are shown for each control compared to the Normal control readings.

	*Normal*	*High intermediate*	*Low intermediate*	*Deficient*
*Number of values*	21	23	25	25
Minimum	111	66	42	2
25% percentile	120	76	53	5
Median	129	84	59	7
75% percentile	138	95	67	9
Maximum	155	101	87	14
Mean	130	84	60	7
Std. deviation	13	11	10	3
Std. error of means	2.9	2.3	2.1	0.5
95% CI	124–136	80–89	55–64	6–8
*Dunnetts multiple comparisons*				
Mean difference	-	46	70	123
95% CI	-	38–53	63–77	116–130

### Comparison of two lots of r-G6PD

**Fig 6:**
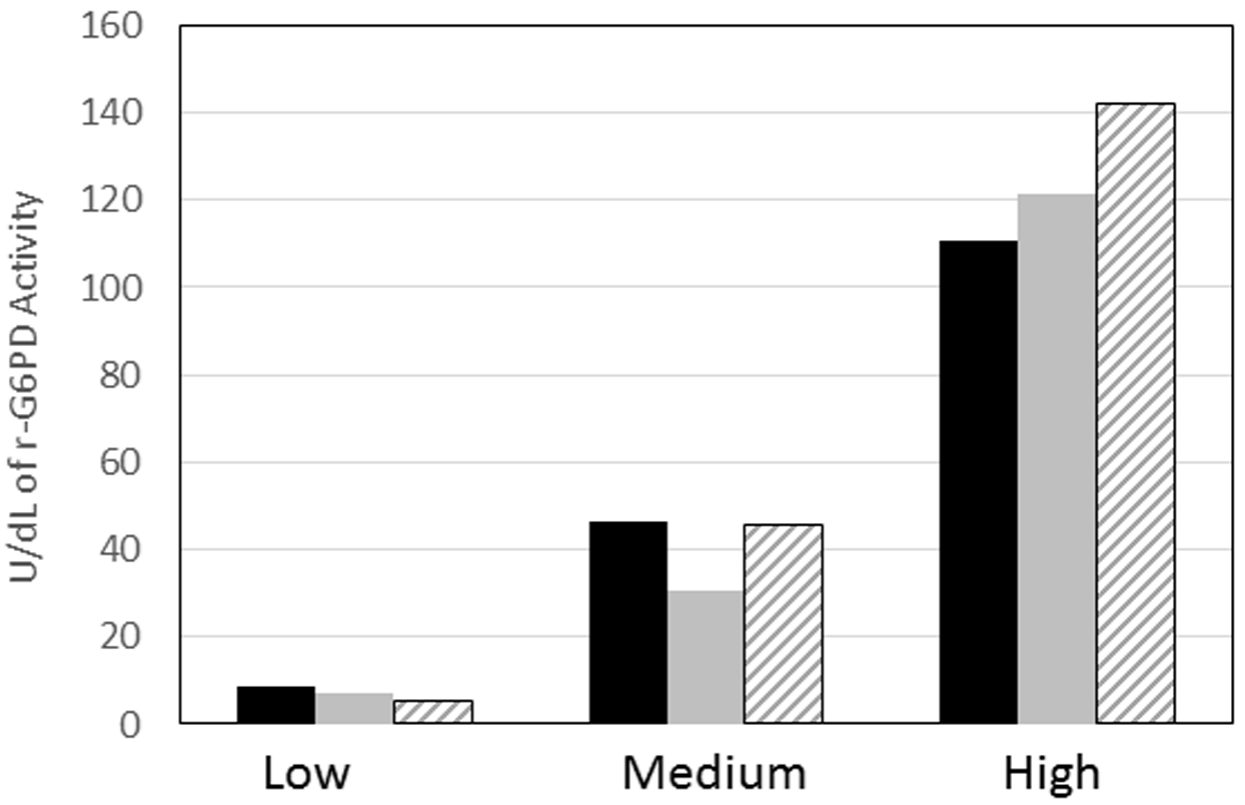
Comparison of freshly lyophilized recombinant glucose-6-phosphate dehydrogenase (r-G6PD) versus year-old lyophilized r-G6PD and control. Human r-G6PD was lyophilized and assayed as “fresh” (grey bars) and compared to lyophilized r-G6PD samples from a lot that had been stored at 4°C for one year (striped bars). The Trinity controls were run at the same time for comparison of activity levels (black bars).

As part of the stability study, two lots of lyophilized r-G6PD were compared; a freshly lyophilized lot of r-G6PD and a lyophilized lot of r-G6PD which was stored at 4°C for one year. The samples were tested using the Trinity Biotech quantitative assay and the Trinity controls were also included. The results in Fig 6 show that there was very little change in activity between the two lots.

**Fig 7:**
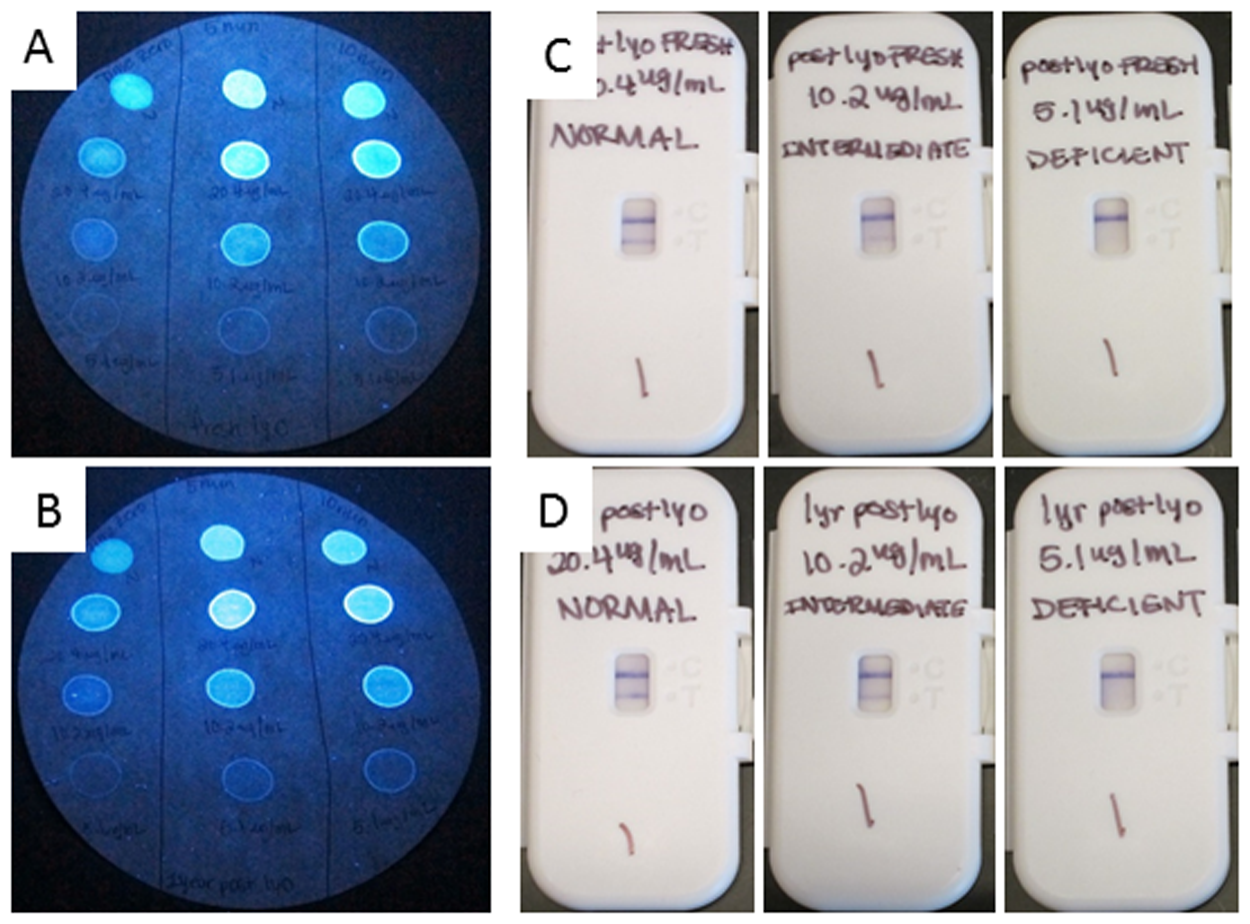
Performance of human recombinant glucose-6-phosphate dehydrogenase (r-G6PD) controls on qualitative tests for G6PD. Two lots of three concentrations (normal, intermediate, and deficient) of human r-G6PD were tested on two qualitative assays for G6PD: the fluorescent spot test (panels A and B) and a novel prototype rapid diagnostic test for G6PD (panels C and D). In panels A and B, the top row represents the Trinity normal controls for reference; the second, third, and fourth rows show the signals for the normal, intermediate, and deficient human r-G6PD controls, respectively. In panels C and D, the line intensity of test output correlates with expected enzyme activity, referring to normal, intermediate, and deficient activity. The signal for one lot of freshly lyophilized human r-G6PD (A and C) is compared to a second lot that had been stored at 4°C for more than one year (B and D).

In addition, the two lots of lyophilized r-G6PD controls were tested using a qualitative G6PD assay, FST, and the newly developed qualitative prototype as described above. These results are shown in Fig 7. The results show that both qualitative assays indicate similar G6PD activity across both lots.

## Discussion

*Plasmodium vivax* infection requires treatment with 8-aminoquinoline–based drugs to eradicate the hypnozoite forms that reside in the liver and cause malaria relapse. Before treatment with 8-aminoquinolines, the status of the enzyme glucose-6-phosphate dehydrogenase (G6PD) should be established. If an individual is severely deficient in G6PD, treating with 8-aminoquinoline–based drugs could result in a hemolytic event and/or death. Assays to detect G6PD that can be run in the field without elaborate equipment will be necessary to optimally treat malaria-infected individuals. Currently, the reliable quantitative G6PD assays require laboratory equipment and skilled clinicians. To assay the G6PD status of individuals quickly and without requiring referral to a high-level clinical laboratory, it is crucial that point-of-care quantitative G6PD assays are available. New point-of-care diagnostic tests, both quantitative and qualitative, are currently being developed to detect G6PD deficiency. These tests will be used to determine which individuals can be treated with 8-aminoquinoline–based drugs. It is important to anticipate the availability of these tests and develop the components necessary to implement robust external quality assurance systems for these products so that they can be implemented by national malaria programs [17, 18].

Feasibility to lyophilize recombinant human G6PD as a quality-control reagent is demonstrated in this article. For calibration of G6PD assays, a standard reagent of human recombinant G6PD was used to create a control panel representing normal, intermediate, and severe G6PD deficiency. This represents 100%, >30%, and 10% enzyme activity, respectively. The stability studies presented here demonstrate that the G6PD enzyme is stable for over a year at temperatures that span working temperatures across a variety of clinical sites.

The controls generated here are multipurpose and can be used to monitor the manufacturing of qualitative point-of-care assays and/or in the determination of whether a point-of-care assay has been compromised. Recombinant G6PD enzyme has been cloned, expressed, and purified and its activity has been determined using the Trinity Biotech quantitative assay for G6PD detection. It has been demonstrated that the lyophilized recombinant G6PD enzyme is stable for at least one year. In addition, it is shown that the lyophilized r-G6PD can be used as a control in a qualitative point-of-care assay for G6PD activity. A target product profile (TPP) that describes how the product will be utilized by the end user [19] for a quality-control reagent that would best support quality assurance of point-of-care G6PD tests was generated (Table 2). Stakeholders from the malaria community as well as developers provided input. Most of the requirements have been met by the formulation described here except for the requirement of shelf-life stability of the rehydrated r-G6PD. All G6PD controls currently available show a short shelf life once reconstituted. The only stable form of hydrated G6PD is when it remains intra-cellular in whole blood and at 4°C [23].

**Table 2:** Target product profile for quality control reagents to support point-of-care diagnostic tests for glucose-6-phosphate dehydrogenase (G6PD) deficiency. Minimal and optimal requirements are described for different product specifications. The unitized recombinant G6PD tubes are expected to meet most specifications with the exception of the shelf-life of the reconstituted reagents (not achieved).

	Minimal requirements	Optimal requirements
**Intended use**	Manufacturer lot release	Clinical laboratory quality assurance (QA) testing, in-country lot testing, staff training for proficiency, support product development
**G6PD activity level panel components**	Deficient (<40%) Normal (>70%)	Deficient (<40%) Intermediate (40%–70%) Normal (>70%)
**Labeling of component levels**	Deficient, Normal	Deficient, Intermediate, Normal
**Preferred frequency of use**	Once per test	Once per day
**Work flow**	Pipette required	Dropper required
**Packaging volumes**	1 quality control (QC) test per vial	5–10 QC tests per vial
**Packaging**	QC reagent and diluent co-packaged	
**Shipping temperature**	4°C	Room temperature (18°C–30°C)
**Shelf life of DRIED reagents**	6–12 months at room temperature (4°C)	6–12 months at room temperature (18°C–30°C)
**Shelf life of RECONSTITUTED reagents**	2–3 months at -20°C (not achieved)	1–3 months at 4°C (not achieved)

The lyophilized r-G6PD has also been shipped at ambient temperatures (in a regular package by courier service) and in excess dry ice by courier service across the continental United States and to the United Kingdom for subsequent testing at Mologic (UK) using G6PD prototypes. The shipping process did not seem to affect the quality of r-G6PD and gave robust results at all three concentrations when tested on a point-of-care G6PD diagnostic. The current formulation meets most of the specifications for a reagent for quality control of qualitative tests for G6PD deficiency. Next steps include modifying the formulation to include hemoglobin in anticipation of the availability of diagnostic tests that measure both G6PD activity and hemoglobin.

## Conclusion

The enzyme glucose-6-phosphate dehydrogenase is available as a stabilized and standardized reference material for researchers, diagnostics developers, and manufacturers of G6PD rapid diagnostic tests. The enzyme can be used for evaluating the performance of G6PD rapid diagnostic tests in quality control programs and for the calibration of G6PD detection assays. While this control is sufficient to be used in qualitative assays, the absence of hemoglobin makes this control currently unsuitable for quantitative G6PD detection. Further studies will be needed to explore the preservation of hemoglobin with the r-G6PD enzyme in a lyophilized format. The combination would make an ideal control that could be used in conjunction with quantitative tests that rely on hemoglobin or the number of red blood cells to determine G6PD activity.
